# Central India Medicinal Plant Dataset (CIMPD)

**DOI:** 10.1016/j.dib.2025.112154

**Published:** 2025-10-09

**Authors:** Rajeev Kumar Singh, Akhilesh Tiwari, Rajendra Kumar Gupta, Satyam Singh Tomar, Muskan, Naresh Kumar Wagri

**Affiliations:** a Department of IT, Madhav Institute of Technology and Science DU, Gwalior, MP 474005, India; b Department of CCS&BM, Madhav Institute of Technology and Science DU, Gwalior, MP 474005, India; c Department of CSE, Madhav Institute of Technology and Science, Gwalior DU, MP 474005, India; d Department of Materials Science and Engineering, School of Industrial Engineering and Management, KTH Royal Institute of Technology, Stockholm SE 10044, Sweden

**Keywords:** Medicinal plant, Plant classification, Leaf images, Image processing, ResNet18, Feature visualization

## Abstract

In the present scenario, medicinal plants play a crucial role in promoting a healthy lifestyle by protecting against numerous diseases. They also hold significant potential as a source of income, particularly for rural populations across the globe. Plants used for herbal medicine are known as medicinal plants, and each part of these plants may be utilized for medicinal purposes. Further, medicinal plants are beneficial in enhancing the human immune system.

In this research, a new medicinal plant named as Central India Medicinal Plant Dataset (CIMPD) has been developed to support significant research in human health. The dataset contains 9130 leaf images (both healthy and unhealthy) from 23 medicinal plant species. These images were collected from various locations in central India. The entire work was carried out over a period of five months, which included plant selection, leaf collection, image capturing, and data organization into folders.

This dataset provides comprehensive information, including the botanical name, common name, geographical origin, healthy and unhealthy leaf images, and medicinal uses of the plants. It serves as a valuable resource for research in machine learning, computer vision, and related domains. Additionally, it will enable the development and evaluation of methodologies for disease detection, plant identification, and other relevant applications.

Specifications Table

**Table:** 

Subject	Computer Science, Agricultural and Biological Science.
Specific subject area	Image classification, Computer vision, Disease detection.
Type of Data	Leaf images
How the data were acquired?	In the proposed work, a fixed setup has been developed for acquiring images. These images are captured using a 64 mega pixel smartphone camera on the Real me X7 features (resolution: 1080×2400 pixels). Camera features (Quad Camera): 64 MP, f/1.8, 266mm(wide), 1/1.7″, 0.8 µm, PDAF, 8 MP, f/23, 119°, 16 mm (ultrawide), ¼.0″, 1.12 µm, 2 MP, f/2.4, (macro), 2 MP, f/2.4, (depth)
Data format	Raw data, RGB, JPEG.
Description of data collection	All collected species are considered as medicinal. Images are captured using fixed-lens smartphone cameras with different configuration, colour variations of leaf and varying distances. This dataset has 9130 images from 23 classes. Within the dataset, there’s an unequal distribution of samples among various classes.
Data source location	For this project, a large no of gardens of various places of central India has visited to collect the medicinal plant leaves.
Data accessibility	Repository name: Kaggle Direct URL to data: https://www.kaggle.com/datasets/satyamtomar08/indian-medicinal-plant-dataset

## 1 Value of the Data

• The development of a medicinal plant dataset plays a crucial role in the exploration of advanced machine learning models for significant investigations such as plant identification, disease detection, crop management, and more [[1], [2], [3], [4]].
• This plant leaf image dataset can also be used to develop new classification models for identifying medicinal plant species (healthy or unhealthy) and addressing various pattern recognition problems [5,6].
• Various image processing techniques such as image segmentation [7], object detection [8], pattern identification [9] can also be employed on presented dataset for numerous research problems.
• This dataset can be utilized to develop educational applications that inform students and raise awareness about Indian medicinal plants and their health benefits [10,11].
• These high-quality plant leaf images can also be utilized for color, texture, and shape analysis, as well as for plant recognition and classification tasks [12].

## 2 Background

The primary aim of developing this medicinal plant dataset is to create a benchmark resource that can be used to address complex research problems [13,14]. This dataset has been specifically designed to facilitate the automated identification of medicinal plants using image processing and machine/deep learning models, thereby simplifying the task and saving valuable time.

An additional objective achieved during dataset development was the capture of images in a controlled environment using a fixed setup. A white paper background and adequate lighting were employed to ensure consistency and clarity in the captured leaf images. Another goal was to streamline the process of medicinal plant identification by providing high-resolution images that are easily analyzable by computational models.

The growing interest in the cultivation and use of medicinal plants has contributed to a wider adoption of optimal health practices among the general population, thereby reducing health risks. A comparative analysis of the presented dataset with other well-known existing datasets is provided in Table 1.

**Table 1:** Comparison of our proposed dataset with existing Indian medicinal plant datasets in terms of species diversity, annotation detail, modality, and application focus.

Dataset Name	No. of Species	Total Images	Annotation Details	Modality	Application Focus
LeafSnap-India	15	7000	Species label only	RGB	Species Identification
MedicinalLeafSet	20	5000	Species + Part annotation (leaf, flower)	RGB	Identification, Educational Use
VNPlant-200	200	40,000	Multi-disease, multi-class	RGB	Disease Detection, Classification
PlantVillage-India	38	54,000	Disease-level annotations	RGB	Disease Diagnosis using DL
Proposed Dataset	23	9130	Leaf-level, Health status (Healthy/Unhealthy)	RGB	Classification, Segmentation

In the current work, the leaf part of the plant has been selected for imaging, as leaves are a reliable feature for plant identification. Not all plants possess distinguishable flowers, fruits, or seeds at all times, but leaves are commonly present and serve as a consistent basis for recognition.

## 3 Data Description

Accurate identification of appropriate medicinal plants is crucial for the effective treatment of various diseases. Traditional (manual) identification methods are often time-consuming and require expert knowledge, making them inefficient for large-scale applications. Therefore, there is a growing need for a novel identification model for medicinal plants. However, the development of such models has been limited due to the lack of comprehensive and well-organized datasets. In this context, the creation of a medicinal plant dataset becomes essential.

The medicinal plant dataset serves as a significant repository for exploring the therapeutic properties of plants and their applications in healthcare. It includes data on various plant species used for their medicinal benefits. The dataset provides detailed information such as scientific names, traditional/common names, geographical origin, number of samples, and the associated medicinal properties of each plant species. This resource can support advanced research and development in the fields of plant science, machine learning, and healthcare informatics.

The dataset also includes additional information, such as the medicinal uses of the plants and specific details about the geographical regions where these plants are commonly found. The process of extracting features from high-quality images is essential for the accurate identification of medicinal plants using computational techniques.

The development of a medicinal plant dataset contributes to the advancement of innovative and sustainable approaches for the utilization and conservation of these valuable natural resources. Medicinal plants have been used for centuries by various cultures around the world for the treatment and prevention of illnesses, and their scientific exploration continues to hold significant potential for modern healthcare applications.

This dataset has 9130 images from 23 different classes of medicinal plant species. Pictures for this dataset were taken in a fixed setup by placing the leaves on a white paper and providing suitable lighting. The dataset consists of approximately 100–110 unique leaf samples, with each image representing a different leaf. No duplicate images or multiple captures of the same leaf (under varying angles or lighting) were used.

**Fig. 1:**
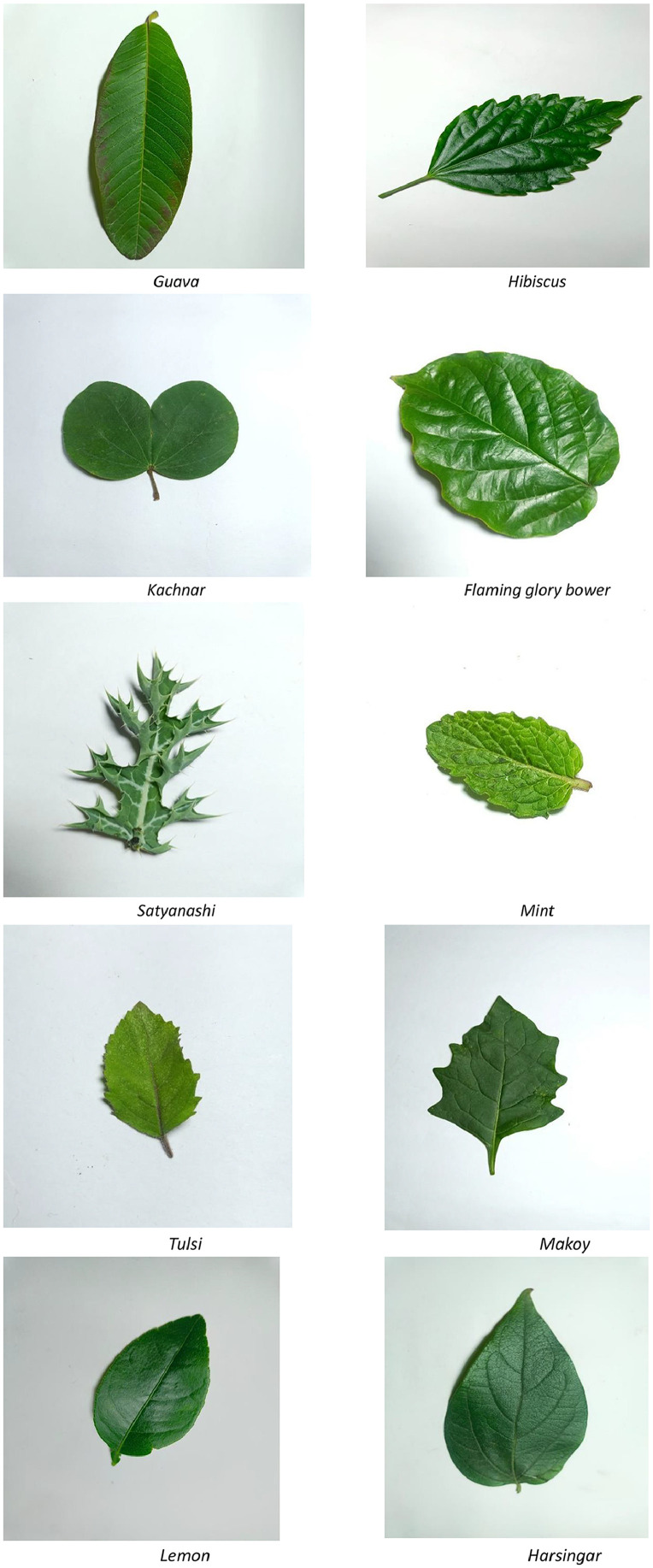
Images of 10 different Medicinal Plants (as sample).

The dataset is organized in a hierarchical folder structure for clarity and ease of use. At the top level, data is divided into three subsets: train**,** validation, and test. Within each subset, images are grouped by plant species, and each species folder is further divided into Healthy and Unhealthy subfolders. The medicinal plants are identified and selected from different regions, location of Gwalior, India including MITS campus. This dataset contains only leaves images. The images of ten medicinal plant leaves as a sample are shown in Fig. 1.The processing time for images is presented in Table 2.

**Table 2:** Time Period for work done.

PROCESS	TIME	WORK
Image Capturing	January to March 2024	For the plant leaf image acquisition, 10,000 images are collected. Out of which, 9130 images are selected for dataset development.
Processing of dataset	April to May 2024	Images are categorised in train, test and validation data through arranging in respective folders in computer system. Feature visualization is done by using Resnet18 model.

The specific details about various plant leaves and other related information i.e. Botanical name, common name, no of species of both healthy and unhealthy leaves are presented in Table 3. Morphological features of various plant species are determined automatically from the Medicinal Plant dataset in Table 4.

**Table 3:** List of Selected Medicinal Plant Species.

SI No.	Common name	Botanical name	Healthy leaf images	Unhealthy leaf images
1	Guava	Psidium gujava	127	62
2	Hibiscus	Hibiscus rosa-sinensis	101	66
3	Lemon	Citrus limon	104	68
4	Rose	Rosa	169	75
5	Tulsi	Ocimum tenuiflorum	355	200
6	Ashoka	Saraca asoca	213	133
7	Harsingar	Nyctanthes arbor-tristis	312	198
8	Marigold	Tagetes	186	–
9	Jackfruit	Artocarpus heterophyllus	211	87
10	Bamboo	Bambusoideae	102	–
11	Nasturtium	Tropaeolum	263	161
12	Barlaria	Barleria cristata	284	–
13	Salvia coccinea	scartlet sage	299	163
14	Kachnar	Bauhinia variegata	327	182
15	Snapdragon	Antirrhinum	172	158
16	Satyanashi	Argemone Mexicana	335	183
17	Flaming glory bower	Clerodendrum splendens	295	198
18	Custard apple	Annona squamosa	367	255
19	Mint	Mentha	240	–
20	Lantana	Lantana camara	456	213
21	Bael	Aegle marmelos Correa	358	201
22	Curry	Murraya koenigii	400	200
23	Makoy	Solanum nigrum	410	241

**Table 4:** Morphological Features of Various Plant Species.

Species	Characters	
	Color	Leave Shape
Psidium gujava	Glossy dark green	Oval or elliptical, with a pointed tip and smooth edges.
Hibiscus rosa-sinensis	Deep green	Ovate or lanceolate, with a pointed tip and serrated edges.
Citrus limon	Vibrant green	Ovate or elliptical, with a slightly serrated margin and a pointed tip.
Rosa	Color ranges from light green to reddish-green	Have an ovate or lanceolate shape.
Ocimum tenuiflorum	Medium to dark green color but in purple varieties, its range from purple-green to deep purple	Oval or lanceolate shape.
Saraca asoca	Dark green	Oblong or lanceolate, with pointed tips and smooth margins.
Nyctanthes arbor-tristis	Glossy dark green	Elliptical to ovate, with a pointed tip and smooth or slightly serrated margins.
Tagetes	Bright to medium green	Lanceolate or ovate.
Artocarpus heterophyllus	Glossy dark green	Broad and oblong with a pointed tip.
Bambusoideae	Vibrant green color	Linear or lanceolate, narrow blades with pointed tips.
Tropaeolum	Bright to medium green color	Rounded or shield-shaped, with a palmate venation.
Barleria cristata	Glossy dark green	Lanceolate to elliptic, with pointed tips and smooth margins.
scartlet sage	Rich green color	Ovate to lanceolate, with a pointed tip and serrated margins.
Bauhinia variegata	Medium to dark green	Bilobed or butterfly shape.
Antirrhinum	Medium to dark margins	Lanceolate or ovate, with a pointed tip.
Argemone Mexicana	Medium to dark green. sometimes also have a bluish green	Deeply lobed and pricky.
Clerodendrum splendens	Medium to dark green	Ovate to elliptical, with a pointed tip, smooth and slightly serrated margins.
Annona squamosa	Glossy dark green	Oblong or oval, with a pointed tip and smooth margins.
Mentha	Vibrant to medium green	Ovate to lanceolate, with a slightly serrated margin.
Lantana camara	Medium to dark green but some leaves have yellow, white or even purple color	Ovate or lanceolate, with serrated margins.
Aegle marmelos Correa	Glossy dark green	Ovate or narrowly elliptical and smooth margins.
Murraya koenigii	Glossy dark green	Pinnately compound.
Solanum nigrum	Medium to dark green	Oval to elliptical, with a pointed tip and smooth margins.

## 4 Experimental Design, Materials and Methods

This section of the experimental design, materials, and methods details all the pre-processing techniques applied to the data to create the final dataset.

### 4.1 Experimental design materials: tools and devices

Images are captured using a 64 mega pixel smartphone camera on the Real me X7 features (resolution: 1080×2400 pixels). Camera features (Quad Camera): 64 MP, f/1.8, 266mm(wide), 1/1.7″, 0.8 µm, PDAF, 8 MP, f/23, 119°, 16 mm (ultrawide), ¼.0″, 1.12 µm, 2 MP, f/2.4, (macro), 2 MP, f/2.4, (depth).

### 4.2 Experimental design

**Fig. 2:**
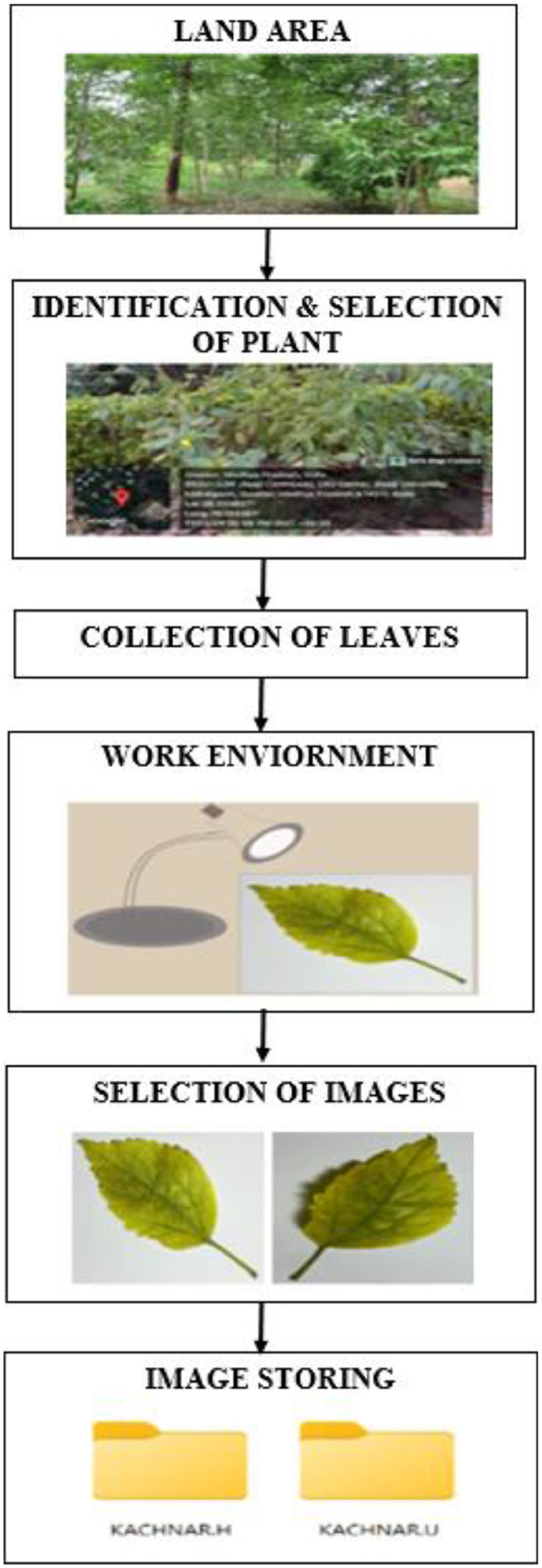
Experimental Work flow.

To collect leaves from various medicinal plants, multiple locations across central India were visited. The images of medicinal plant leaves were captured between January and March 2024 using a mobile phone equipped with a 64-megapixel camera. The complete experimental workflow is illustrated in Fig. 2.

### 4.3 Methods

In the present work, the PyTorch framework is employed. PyTorch is an open-source machine learning library based on the Torch library, widely used for applications in computer vision and natural language processing. We utilized the ResNet18 model, which is pretrained on the ImageNet dataset. Prior to feeding the images into the model, all input images were resized to ensure uniform dimensions, as convolutional neural networks (CNNs) require input data in the form of tensors. Therefore, the images were transformed into tensors accordingly.

**Fig. 3:**
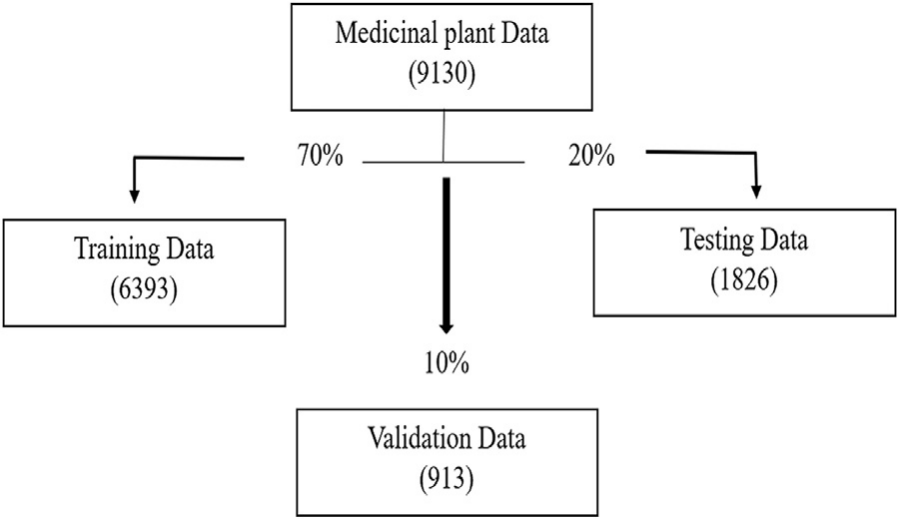
Data Distribution.

The distribution of images across the dataset is illustrated in Fig. 3, where a total of 9130 images are divided into three subsets: 6393 for training, 1826 for testing, and 913 for validation. The class-wise label distribution, categorized by plant species and health status (Healthy/Unhealthy), is detailed in Table 3 Following dataset preparation, the Conv2d layers of the ResNet18 model were extracted and stored in a list along with their corresponding weights. After applying the model to the dataset, a total of 17 convolutional layers were extracted. Table 5 visualizes the features extracted by the first, fourth, and seventh layers of the Resnet18 model.

**Table 5:** Feature Visualization.

## Limitations

The dataset may be limited by device dependency (smartphone camera), seasonal/regional sampling bias, and class imbalance across plant species. These factors could affect model generalizability and performance in diverse real-world conditions.

## Ethics Statement

This study does not involve any human participants or animal experiments. It contains photographs only. All the photographs were captured by the author and does not include any images that were gathered from any other site. This research was conducted without any financial support and grants.

## Credit Author Statement

**Rajeev Kumar Singh:** Conceptualization, Supervision, Methodology, Writing - original draft; **Akhilesh Tiwari:** Supervision, Writing - review; **Rajendra Kumar Gupta:** Supervision, Writing - review & editing; **Satyam Toma**r**:** Data curation, Writing – original draft; **Muskan:** Data curation, Writing - original draft; **Naresh Kumar Wagri:** Review & Editing

## Data Availability

KaggleCentral India Medicinal Plant Dataset (CIMPD) (Original data).
